# Grand Challenges: Integrating Mental Health Services into Priority Health Care Platforms

**DOI:** 10.1371/journal.pmed.1001448

**Published:** 2013-05-28

**Authors:** Vikram Patel, Gary S. Belkin, Arun Chockalingam, Janice Cooper, Shekhar Saxena, Jürgen Unützer

**Affiliations:** 1 Centre for Global Mental Health, London School of Hygiene & Tropical Medicine, United Kingdom; 2 Sangath, Goa, India; 3 Centre for Mental Health, Public Health Foundation of India, India; 4 Program in Global Mental Health, New York University School of Medicine, New York, United States of America; 5 New York City Health and Hospitals Corporation, New York, United States of America; 6 Simon Fraser University, Burnaby, Canada; 7 The Carter Center Mental Health Liberia Program, Rollins School of Public Health, Emory University, United States of America; 8 College of Science & Technology, University of Liberia, Monrovia, Liberia; 9 Department of Mental Health and Substance Abuse, World Health Organization, Switzerland; 10 Division of Integrated Care and Public Health, Psychiatry and Behavioral Sciences, University of Washington, Seattle, United States of America

## Abstract

In the last article of a five-part series providing a global perspective on integrating mental health, Vikram Patel and colleagues discuss the competencies, operational innovation, and packages of care needed, and argue that integration will complement primary care system strengthening.

*Please see later in the article for the Editors' Summary*

**Summary Points**

- The rationale for integration of mental health care into other health care platforms includes improving access to mental health care; providing patient-centered care; avoiding fragmentation of health services; reducing stigma; optimising both mental health and physical health outcomes; and overall health system strengthening.
- Interventions for common mental disorders and alcohol abuse, the mental disorders contributing to the greatest global burden of disease, are the most promising for integration.
- The process of integration requires assessment of and customization for the specific platform; identification of tasks and human resources for case finding and delivery of interventions; and application of the principles of collaborative care, care management, and quality improvement.
- The risks of a purely integrated approach to mental health care are that some types of mental disorders may be neglected, that there might be an overburdening of already weak health systems, and that the evidence base for scaling up of integrated interventions is patchy.
- Integrated care is smart because the operational and functional innovations needed for such integration into other health care platforms are consistent with efforts to strengthen the capacity of primary care systems to address multiple health priorities more broadly.
- This paper is the fifth in a series of five articles providing a global perspective on integrating mental health.

*This is one article in a five-part series providing a global perspective on integrating mental health.*

## Introduction

In this final paper in a five-part series highlighting the opportunities for integrating mental health care into priority global health programs and platforms of health service delivery, we aim to synthesize the evidence presented in the articles in the series addressing maternal health [1], non-communicable diseases (NCD) [2], and HIV/AIDS care [3], with the goal of identifying overarching themes across these platforms [4]. Our focus is on competencies and work packages appropriate for health care settings that do not historically address mental health issues and that do not usually include mental health specialists. Primary health care is the quintessential example of such a care delivery platform. In this paper, we consider the rationale for integration, the extent to which specific mental disorders can be addressed in other delivery platforms (and, the corollary, which disorders may need a more specialized approach to care), the process of integration, potential risks and barriers to successful integration and strategies how these might be addressed, and the promise of this approach for addressing the leading Grand Challenges in Global Mental Health [5].

## Why Integration

Mental health problems, such as depression, anxiety, and alcohol and drug abuse, are among the most common and disabling health conditions worldwide [6]. They often co-occur with acute and chronic medical problems and can substantially worsen associated health outcomes [7]. When mental health problems are not effectively treated, they can impair self-care and adherence to medical and mental health treatments, and are associated with increased morbidity and mortality, increased health care costs, and decreased productivity.

Effective treatments exist for most common mental health problems [8], but few patients have access to such treatments. Adequate access to mental health specialists is a challenge, especially in low- and middle-income countries (LMICs). For example, the number of psychiatrists serving the entire continent of Africa with a population of almost a billion is less than that practicing in the US state of Massachusetts with a population of less than 7 million. But even in developed countries ,such as the US, , primary care practices are the de facto location of care for most individuals with common mental health problems [9] and only 2 in 10 adults with common mental health problems receive care from a mental health specialist in any given year [10]. To reach a reasonable proportion of community-living individuals with common mental health problems will require leveraging the limited number of mental health specialists as consultants to help enhance the capacity of primary care and other care delivery settings that do not provide specialty mental health services to address these common problems.

There are at least two additional advantages to treating common mental health problems in primary care and other priority health care programs. First, integrated treatment programs in which medical providers are supported to treat common mental health problems offer a chance to treat ‘the whole patient’, an approach that is more patient-centered and often more effective than an approach in which mental health, acute and chronic physical health, reproductive health, and chronic pain problems are each addressed in a different ‘silo’ without effective communication between providers. Second, integrated care programs that can address patients' mental health needs in the context of general or other specialized health care settings are often more attractive to patients and family members who are concerned about the stigma that is still associated with mental and substance abuse disorders and the treatment settings that specialize on caring for individuals with severe mental disorders.

Treatment of common mental health problems in primary care can be improved via evidence-based collaborative care interventions, yielding better access to care, better physical as well as mental health outcomes, and improved overall cost-effectiveness [11]–[14]. An integrated approach to addressing mental health in the context of care for HIV, maternal mental health, and NCD is rooted in the conviction and growing evidence of its efficiency, effectiveness, and cost-savings [15]. Prevention and early intervention also contribute significantly to reducing the global burden of disease, both mental and physical. An integrated population-based approach that seeks to prevent conditions affecting mental health and physical health would share many common strategies; for example, motivating behavior changes, such as reducing alcohol intake and smoking; promoting physically active lifestyles; and restricting the sale and distribution of tobacco and alcohol products [16]. Finally, integrating mental health can accelerate progress and achievement of sustainable development goals by leveraging existing health platforms designed to care for individuals with HIV/AIDS and other health problems [17].

## What to Integrate

As suggested in other articles in this series [1]–[3], the proposal is to integrate care for common mental health problems into the routine care for people affected by other chronic NCD (such as cancer, diabetes and cardiovascular disorders), HIV/AIDS, and maternal health care. Collectively, these health care contexts address the lion's share of the global burden of disease. A common theme that runs through all of these delivery platforms is that two types of mental health conditions are particularly ripe for integration given their prevalence and evidence as to the effectiveness of “task-shared” care: common mental disorders, such as depression and anxiety; and alcohol use disorders. The responses to these conditions share common core elements, including implications across the lifespan, strong association with poverty and education, potential for prevention and early intervention, multiple points for identification and treatment, the need for a collaborative approach to care, and the availability of pharmacological and psychosocial treatments that can be delivered by non-specialists with adequate support and have potential for strong stakeholder involvement [18]. Effective integration efforts should include workforce development and capacity building supported by training guidelines for clinical and psychosocial management of care; effective tools, such as screeners and validated instruments to track clinical outcomes; consumer and family support; and policies and payment systems supportive of integrated practice. They should also include routine and effective use of outcomes monitoring, evaluation, and research to recognize effective practices [4].

## How to Integrate

### Assessment and Customization

Because there are wide variations in the capacity and readiness of priority health care programs within countries, adequate assessment and customization is essential for planning the integration of mental health care. An example is the Integrated Management of Adult and Adolescent Illnesses (IMAI) used in providing mental health care to persons with HIV/AIDS (http://www.who.int/3by5/capacity/fs/en/index.html). Joint assessment by the managers of the priority health programs and mental health professionals/service planners also enhances ownership and commitment to achieve the planned outcomes within agreed timelines. The most common reasons for failure to integrate mental health care into primary or other priority health care programs are lack of adequate assessment and overly ambitious target setting without the necessary customization of the detailed activities, and a full and explicit agreement on the targets and activities needed to achieve them. The following steps may facilitate optimization of the integration.

- Assessment of the goals, functions, and resources (human and financial) of the priority program. This step should include attention to the existing knowledge and skills of health care providers as relates to their identification and care of common mental health problems; recognition of when to refer; inclination/motivation to enhance their skills; and the perceived benefits of these skills to advance their professional and programmatic goals. For example, stakeholders need to agree that mental health treatment within maternal and child health platforms advances specific Millennium Development Goals and front line clinicians must see the value of adding these treatments to their current services.
- Identifying shared and achievable objectives. This step requires joint assessment of the needs and feasibility of integration; the identification of exact tasks; and the training, support, and supervision needed for clinicians to provide these services. Attention must be paid to congruence of the integration efforts with the overall objectives of the priority programs and the resources needed to ensure initial success and sustainability. Beginning with limited but clear and specific objectives is recommended. For example, the initial target for integration of mental health care within HIV programs may be the identification and management of depression to achieve better adherence with HIV care.
- Assigning responsibilities and establishing a monitoring mechanism. Clear and explicit responsibilities need to be assigned to the health care providers and managers of the priority programs and to the mental health team at each level. Flowcharts and referral algorithms, such as WHO's mental health Gap Action Programme (mhGAP)-Intervention guide [8], can be very helpful in this step of planning. . They also can then be linked to the monitoring mechanism using a limited number of clear, relevant, and agreed-on goals.

### Tasks and Human Resources

The papers in this series have emphasized the importance of preventing, identifying, and reducing the burdens of co-occurring *disorders* for population health [1]–[3]. But the key challenge facing scale up of all health care is the effective deployment of complementary *skill sets* in order to address a range of health problems within a shared platform. Such co-competency needs as much attention as co-morbidity. A substantial obstacle to the integration of mental health care is lack of consensus over how to standardize and assign mental health care tasks so they can be scaled up within overall delivery. Consensus treatment packages, such as those in the WHO mhGAP-Intervention Guide, describe what counts as good and evidence-based care [8]. But these packages need to be adapted and integrated into existing health care systems. For any health workforce to be effective, and for care packages to be delivered as intended, treatment guidelines need to be operationalized into coordinated roles and tasks. The starting point for effective integrated care pathways is to specify skill sets necessary to effectively deliver integrated care and plan for the development and deployment of these skills in the context of available human resources. Building blocks for such core skill sets include: (1) screening, engagement, education of patients and family members, close follow-up, and tracking of adherence and clinical outcomes; (2) targeted, evidence-based psychological interventions (e.g., motivational interviewing, behavioral activation, problem-solving or interpersonal therapy); (3) pharmacologic treatment; (4) population-based outcomes tracking and quality improvement; and (5) specialist supervision and consultation [19].

Effective treatment programs bundle skills that logically group together in terms of content, needed training, and operational use. Most of the required functions can be performed by a range of workers, most of whom are already part of primary care settings, thus allowing some flexibility in planning and adaptation and marginal additional investments. Experience with task-shifting and/or task sharing, as highlighted in the case studies in other articles this series [1]–[3] shows that many of the required skills and tasks of care can be learned and delivered by a range of non-specialist health workers with appropriate training and supervision. Particular skills, such as case finding, support of treatment adherence and motivational coaching, follow-up tracking, patient education, and self-management support, turn out to be quite critical to providing effective care. These “care management tasks” or work packages can be effectively assigned to non-specialist health workers who are well positioned to bring them into the community, extending the reach of primary care.

Several recent meta-analyses have concluded that Collaborative Care (CC), the best-evaluated model for treating common mental disorders such as depression or anxiety in primary care settings, is consistently more effective than care as usual [13],[20],[21] (Table 1). CC builds on the foundations of effective collaborative management of other chronic diseases, such as diabetes. Katon and colleagues recently reported on the effectiveness of a TEAMcare (http://www.teamcarehealth.org/) approach in which nurses and consulting specialists support primary care providers in successfully managing multiple chronic diseases including depression, diabetes, and heart disease [22]. This example underscores how innovation for integrated mental health care can align with and accelerate overall health systems strengthening. While much of the CC evidence is based on research literature from high-income countries, such as the United States, Canada, the United Kingdom, and the Netherlands, there is a growing evidence base testifying to its applicability in primary care in LMIC [23],[24]. The papers in this series show that the components summarized in Table 1 are also effective and feasible in LMIC [1]–[3]. Several randomized controlled trials show that lay community health workers and nurses can effectively provide depression management in low-resource settings, including such psychotherapies as interpersonal psychotherapy [23],[25], cognitive behavioral therapy [26],[27], behavioral activation [28], and problem-solving therapy [29], as well as medication monitoring and management [23]. The MANAS trial in Goa, India brought many of these elements together in an effective package [30].

**Table 1:** Key Elements of Collaborative Care for Depression.

Component	Description of Features
Self care support	Patient/family education about illness and treatments, self-monitoring, management, and adherence support and skills
Care management	Monitor adherence, side effects, change in symptoms, and course of care following evidence-based guidelines.
Treatment to target	Systematic tracking of depression severity and treatment adjustment/intensification aimed for patients not improving as expected following evidence-based treatment algorithms
Systematic caseload review, consultation, and referral	Regular review with a specialist (in person or remotely) of all patients in a caseload not improving as expected. Consulting specialist makes recommendations for treatment changes and/or referral to more specialized services as needed.
Case registry	Use of a registry to track clinical outcomes (e.g., depression severity scores) and key process steps and to facilitate transparent shared management across non-specialist workers, primary care providers, and consulting specialists
Proven intervention strategies	Use of evidence-based interventions (e.g., medication management or psychological treatment strategies) that are supportable by available skill level and consistent with mhGAP-IG

### Standardization

CC is amenable to the kind of standardization needed for scaled integration because it follows the principles of measurement-based care [31], treatment-to-target, stepped care [32], and other aspects of the chronic illness care model proposed by Wagner and colleagues [33]. In such programs, each patient's progress is closely tracked using validated clinical rating scales (e.g., the Patient Health Questionnaire-9 (PHQ-9) for depression [34]), which is analogous to how patients with diabetes are monitored via HbA1c laboratory tests. Treatment is systematically adjusted — “stepped” up — if patients are not improving as expected with input from a specialist consultant. Patients who continue to show no response to treatment, or have an acute crisis, are referred to mental health specialty care; in practice, however, only a relatively small fraction of patients in CC programs request or require this referral. Such systematic ‘treatment to target’ can prevent patients from falling through the cracks and overcome the clinical inertia that is often responsible for ineffective treatments of common mental disorders in primary care [35].

The systematic implementation of evidence-based CC programs challenges the conventional wisdom that while physical health skills are objective, mental health skills are highly subjective and so are not amenable to standardization. A workflow description, or care pathway, aligns and connects these CC elements, matching roles with the appropriate skill sets and triage decisions, and application of screening or symptom tracking tools. This approach positions and leverages more specialized clinical judgment at the right stage of care. The Partners in Health/Zanmi Lasante health system in Haiti, for example, after listing key skill packages and assigning them across available workers, adapted the CC key elements into a care pathway for integrating depression care that maps out standard work and triage points, supported with a locally validated symptom scale [32]. Effective development and implementation of integrated care pathways and routines, and their successful scale up, require ongoing, iterative adaptation, hypothesis testing, performance data monitoring, and improvement [19].

Proven quality improvement (QI) methods have been shown to be effective in LMIC for sustained scale up and adaptation of standardized treatment packages for Millennium Development Goal health priority areas. There is growing acceptance of and attention to quality improvement as a critical part of health systems strengthening for health in LMIC. Quality improvement should also be a routine part of mental health implementation and customization in these settings [36].

## Limitations and Potential Risks

A key limitation to the proposal to integrate mental disorders is the relatively uneven evidence base existing across platforms of care and the almost complete absence of evaluations of scaled-up integrated care programs outside high-income countries (HIC) needed to guide the process [37]. Other papers in this series show that while there is a reasonable evidence base, in the form of randomized controlled trials, on the integration of interventions for depression in maternal health programs in LMIC [1], the evidence base for HIV/AIDS is weaker [3], and such evidence is completely absent for NCD or for integrating care for other mental disorders from LMIC [2]. From a global perspective, however, including the overall evidence base in support of integration, including evidence from high income countries, is more compelling.

Health care systems vary in their ability to respond to national health care needs. As Samb and colleagues point out, a robust approach to addressing mental health conditions, HIV, or NCD requires strong health systems [38]. Many health care systems, and particularly those in fragile post-conflict settings, lack the core health system elements needed to provide the most basic set of services to address mental health, chronic conditions, or HIV/AIDS [39]. Problems include poor financing and a fiscal infrastructure largely dependent on external aid, fragmentation of structures and services, weak systems for procurement (including inadequate supply of medications and poor or no access to diagnostic services), inadequate or fledgling governance and leadership [40], and a workforce that is often overwhelmed and experiencing high turnover. Integration may be the only feasible option to address mental health problems in the context of a weak health system, and doing so can contribute to systems strengthening more generally. Meeting mental health needs, as has been argued is this series, involves precisely the kinds of delivery design innovations needed for overall system strengthening and development. Such a route has a proven, albeit limited, track record, and getting there will need alignment of objectives between donors and governments, a “sector wide” approach to health care, and secured new investments [40].

A final and important concern about the goal of integration is the scope of mental disorders that are suitable for integrated care. The papers in this series do not address the important burdens of severe and persistent mental disorders, such as chronic psychoses; childhood mental disorders, such as autism; or neuropsychiatric disorders, such as epilepsy, dementia, or the neuropsychiatric sequelae of traumatic brain injuries. These disorders, put together, account for at least half of the overall burden of mental disorders. The lack of evidence on integrating care for these disorders with routine platforms — for example, child health care for child mental disorders — is not in itself an indicator that such integration is not feasible, but instead, that this represents a priority research agenda. Other concerns may involve the potential diversion of scarce mental health resources from individuals suffering with severe, chronic psychotic disorders to individuals with less severe common disorders, such as depression and anxiety, seen in primary care settings.

## Next Steps

Integration of care is smart because of the impact of untreated mental disorders on the course, risks, and outcomes of other health conditions. Integration of care is the only feasible way to provide care for mental disorders in most LMIC (Box 1). An equally important message it that integrated care is smart because the operational and functional innovations needed for such integration into other health care platforms are consistent with efforts to strengthen the capacity of primary care systems to care for individuals with multiple health problems more broadly. Thinking in this integrated way about systems strengthening will therefore also position health systems to contribute to solutions that improve population well-being. This is a broader, multi-sectoral framing of health and social development that will require operational capabilities to integrate interacting social and clinical determinants of overall health and functioning.

**Box 1. Next Steps**

- Integrate mental health into routine health care platforms, because this integration is the only feasible way to address the treatment gaps for mental disorders, in particular for common mental and alcohol use disorders.
- Use proven methods of care management, supervision, support, and evaluation, which provide a robust starting point and common framework for implementation of integrated mental health care.
- Conduct further implementation research to build the evidence base on integration and scaling up of care for mental disorders in routine health care platforms.
- Refine the skills packages for various members of the health workforce.
- Explore the integration of a wider set of mental disorders, such as severe and child mental disorders, in routine health care platforms.

Scaling up of the evidence presented in this series will greatly benefit from further implementation research. Trials and other types of evaluation studies are needed, for example, to test the applicability in LMIC of multi-disease CC as demonstrated by Katon in a high-income setting [22]. The evidence base in the form of trials of integrated interventions may be greatly enhanced as a result of new funding for such experiments (such as the National Institute of Mental Health Hubs and R01 RFAs and the Grand Challenges Canada), programs seeking to evaluate scaled up mental health programs in LMIC (such as the United Kingdom's Department for International Development (DFID) -funded PRIME consortium; www.prime.uct.ac.za), and new avenues for publication of mental health integration in practice in this journal [41] amongst others. Key elements in these programs would be further refinement of skills packages for various members of the health workforce and an exploration of the integration of a wider set of mental disorders in routine care platforms. Expanding the integration agenda to address child mental disorders (for example, in school and paediatric care platforms), epilepsy, and the prevention of mental disorders are important priorities for future action. We urge Health Ministries and researchers alike to understand that skill package-based planning and CC, as well as the use of proven methods of supervision, support and evaluation, provide a robust starting point and a shared language and framework for implementation of integrated mental health care in LMIC.

## Abbreviations

CC: Collaborative Care
LMIC: low- and middle-income countries
NCD: non-communicable diseases
